# A Review of Thermal Spectral Imaging Methods for Monitoring High-Temperature Molten Material Streams

**DOI:** 10.3390/s23031130

**Published:** 2023-01-18

**Authors:** Katarina Grujić

**Affiliations:** NORCE Norwegian Research Centre AS, 4630 Kristiansand, Norway; kagr@norceresearch.no

**Keywords:** spectral imaging, metallurgy, infrared thermography, pyrometry, temperature measurement, flow rate

## Abstract

Real-time closed-loop control of metallurgical processes is still in its infancy, mostly based on simple models and limited sensor data and challenged by extreme temperature and harsh process conditions. Contact-free thermal imaging-based measurement approaches thus appear to be particularly suitable for process monitoring. With the potential to generate vast amounts of accurate data in real time and combined with artificial intelligence methods to enable real-time analysis and integration of expert knowledge, thermal spectral imaging is identified as a promising method offering more robust and accurate identification of key parameters, such as surface temperature, morphology, composition, and flow rate.

## 1. Introduction

Metals are a highly valuable resource in modern industrialized societies, and the demand is only expected to increase in the future. Metal production produces a substantial effect on the environment through depletion of non-renewable natural resources and extensive energy consumption, leaving a considerable carbon footprint and polluting the environment. Further to this, various high-purity metals are heavily intertwined in parts of almost every consumer product. This significantly complicates the end-of-life treatment and limits both the quantities and qualities of materials recovered through recycling processes. To be able to move away from the linear “take-make-use-dispose” economic model and towards extending the products’ lifecycle and finally to achieve a sustainable, circular economy, new technical skills for recycling and recovery of metals must be developed [1]. The variability of products and materials demands a dynamically agile metallurgical processing infrastructure capable of absorbing and processing into high-quality products and large amounts of raw material composed of complex recyclates mixtures [2]. This can only be enabled by connecting all stakeholders through digital integration of metallurgical reactor technologies and systems, which in turn requires the development, calibration, and integration of dynamic simulation models of the different processes [3]. Metal smelting is particularly important as a fundamental process in pyrometallurgical metal production, occurring in different types of reactors, such as blast and electric arc furnaces, and involving temperatures ranging from 600 °C to as high as 3000 °C [4,5,6]. The core of such a metallurgical reactor is beyond the reach of existing sensor technologies due to the prevailing extreme temperatures, aggressive chemical environment, and large electric currents. Insights into the state of the process can be obtained through computational modeling. A large number of models has been developed for selected furnace components or features, such as the electrodes [7], electric arcs [8], tapping, and gassing [9]. By combining the validated sub-models and simulations, we can indeed understand these processes and make them more transparent, comprehensible, and safe [10]. By integrating data from various sources, hybrid digital twins of metal smelting processes can be developed to recognize, forecast, and communicate in advance the less optimal behavior of their physical counterparts [11]. However, the extreme metallurgical process conditions suffer from a lack of data to validate the modeling results [6]. At this point, many of the defining properties are hardly measurable, with large variances. Measurements of temperature, levels, pressure, and flow rates, often trivial at lower temperatures, are either not possible or cannot be simply performed at extreme temperatures [12]. Insights into metal smelting process reactors are thus limited, and there is still an unmet demand for real-time feedback to allow consistent and high-quality production. As an alternative, indirect way of learning about the conditions at the furnace heart, more accessible processes have been studied. The process of furnace tapping is a common process directly influenced by in-furnace governing conditions and furnace operation [13,14,15]. The molten material pouring stream is directly open to the environment and accessible to different monitoring systems. Nevertheless, accurate measurements still present considerable challenges. Installation of sensors on the vessel itself or in the tapping zone is often inconvenient and may present durability problems. Any contact measurement requiring direct human intervention close to the hot material is potentially dangerous, e.g., temperature measurement in the furnace tapping stream by using a lance with a thermocouple. As a consequence, non-contact measurement methods have attracted a great deal of attention, such as optical pyrometry and thermal imaging, as they are capable of contact-free scanning of large areas in a short time. However, for users of thermographic equipment without in-depth knowledge of radiative heat transfer principles and sufficient understanding of thermal imaging methods, correct interpretation of results may prove difficult, especially in case of measurements in dynamically changing environments that involve targets with varying radiative properties, such as molten material pouring streams. 

Foreseeing a need for development of more accurate, real-time sensing solutions for furnace tapping processes in the future, this paper presents a review of the state-of-the-art of the different thermal imaging approaches limited to the methods suitable for highly dynamic targets with changing radiative properties. The paper presents an overview of the radiation thermometry working principles in Section 2. The basic concepts of thermal imaging are briefly presented in Section 3 as a starting point for further explorations. The differences between the traditional broadband thermal detectors, such as microbolometers and wavelength-sensitive, spectral imaging sensors are emphasized. Finally, in Section 4, applications of the different methods are reviewed, and strengths and limitations discussed with respect to monitoring key properties of molten material pouring streams: detection of slag carry-over, measurement of surface temperature, and characterization of surface composition, turbulence, and flow rate. 

## 2. Radiation Thermometry Working Principles

The radiometric measurement of temperature is of great practical importance and is widely used in science and industry when the conventional temperature measurement by thermocouples, resistance thermometers, or other sensors are found to be unsuitable. It is based on the fact that, as a consequence of its temperature, all matter emits thermal radiation in the intermediate portion of the electromagnetic spectrum, which includes a portion of the ultraviolet and all of the visible and infrared wavelengths. For liquids and solids, radiation is a surface phenomenon, and the magnitude of radiation at any wavelength will vary with temperature and the nature of the emitting surface. A surface may emit radiation preferentially in certain directions although many surfaces can be reasonably approximated as isotopically diffuse emitters, emitting radiation equally in all directions. Radiation thermometers, otherwise known as pyrometers, sense radiant flux from the target; that is, the total radiant power emitted from the source of radiation that reaches the photodetector via an optical system that defines the thermometers field of view. 

Most commercial radiation thermometers are direct-reading with the calibration algorithm embodied in the electronics. The calibration is performed by sighting the instrument upon a blackbody at a known temperature. A blackbody is a surface of zero reflectivity, i.e., an ideal radiator. When cool and observed at visible wavelengths, it appears black since, at room temperature, most of the emission occurs in the infrared. When observed at infrared wavelengths, blackbody appears brighter than its surroundings and equally bright no matter what material it is fashioned from. A blackbody has the advantage of a known relationship between its spectral radiance, $L_{bb,\lambda }$, and temperature [16]. This relationship is known as the Planck’s law:

(1) $$L_{bb,\lambda }=2hc\lambda^{-5}\frac{1}{\exp\left(\frac{hc}{\lambda kT}\right)-1},$$

where h is Planck’s constant, c is the speed of light, k is Boltzmann’s constant, T is the absolute temperature, and λ is wavelength. 

**Figure 1:**
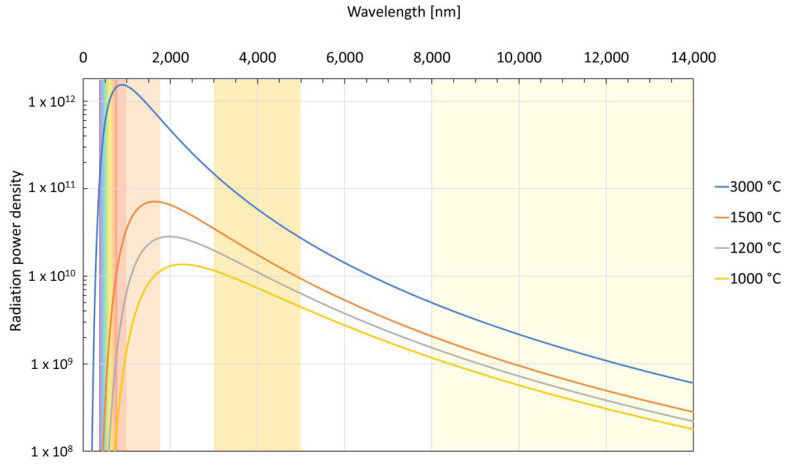
Blackbody radiation is a function of temperature. High-temperature objects emit most of the radiation in the near- to mid-infrared. The infrared spectral range is usually divided in visible spectrum (VIS, 380–750 nm), near-infrared (NIR, 750 nm–1 µm), short-wavelength infrared (SWIR, 1–1.7 µm), mid-wavelength infrared (MWIR, 3–5 µm), and long-wavelength infrared (LWIR, 8–14 µm).

A detector receives the radiant energy focused on it by the instrument’s optical system and generates an electric output signal in response to it. In order to suit different applications, a range of pyrometers has been developed to operate in a variety of wavelength bands (see Figure 1). The choice of the wavelength is governed by the opacity of the target and the atmospheric absorption properties for any given application. An instrument using a near-infrared (NIR) band can measure high temperatures, 700–3600 °C, while instruments operating in long-wavelength infrared (LWIR) can measure down to −45 °C. The instruments operating in NIR and short-wavelength infrared (SWIR) are used for metals. Glass is transparent to shorter wavelengths, and measurement can thus be taken through quartz windows. At longer wavelengths, glass is opaque, so the optics must be fabricated from MWIR/LWIR transmitting optical materials, e.g., germanium. Such optics are custom-made to ensure good imaging properties and thus very expensive, especially when compared to glass optics with similar imaging capabilities to be used in Si charge-coupled device (CCD) video cameras. Thermal detectors, e.g., thermistor-type bolometers, measure through heating of the material with a temperature-dependent resistance [17]. Since the absorption mechanism is thermal and not photonic, there is no cut-off wavelength, and true broad band sensitivity may be achieved. Relative to photodetectors, these devices have lower sensitivity and a speed of response limited to a 10–15 ms range [18]. Photodetectors, on the other hand, are wavelength-sensitive, with response times in the order of microseconds. Their main disadvantage is instability at longer wavelengths and higher temperatures, often requiring cooling.

### 2.1. Spectral Emissivity and the Apparent Temperature

In the temperature measurement process, the radiation thermometer’s output, referred to as the apparent temperature, is a signal that, through the calibration algorithm, corresponds to the temperature of an equivalent blackbody. In practice, the target seldom approximates a blackbody radiator, so to describe the emission from a real surface, we define the surface radiative property, namely spectral emissivity, $\varepsilon_{\lambda }$, as the ratio of the spectral radiance emitted by the surface to that of a blackbody at the same temperature, viewed in an identical manner. The emissivity of a surface depends on the emissivity of the material from which it is made but also on the surface roughness, degree of oxidation, and shape. Surfaces whose emissivity is less than unity but constant at all wavelengths are called graybodies. For thermometry applications, a target may be treated as a graybody if its emissivity is constant for all wavelengths to which the radiation thermometer is sensitive. Furthermore, the target environment, comprised of its surroundings and the atmosphere in the line of sight, is not well-controlled as during the calibration process. This affects the received radiation, which is comprised of emission from a non-black target, reflection of irradiation originating from the hotter surroundings, and finally, absorption and emission by atmosphere in the line of sight. To relate the apparent temperature to the true temperature of the target surface, we thus need information about both the target surface and its environment. 

For a freely radiating target in cool, non-reflective surroundings with no participating atmosphere, in many cases of practical interest, Planck’s law can be simplified through Wien’s approximation. It can be used with little loss of accuracy (<1%) where λT<3000 µm K. This leads to a relationship that simply relates the true surface temperature ($T_{s}$) to the apparent temperature ($T_{a}$) from the knowledge of the spectral emissivity and the mean effective wavelength [16]:

(2) $$\frac{1}{T_{s}}=\frac{1}{T_{a}\left(\lambda \right)}+\frac{\lambda k}{hc}\ln\left(\varepsilon_{\lambda }\right).$$

This relationship is the basis of the narrow-band or spectral radiation thermometer. The uncertainty in the temperature resulting from an uncertainty in knowledge of the spectral emissivity for an observed apparent temperature can be assessed from

(3) $$dT_{s}/T_{s}=\frac{\lambda kT}{hc}d\varepsilon_{\lambda }/\varepsilon_{\lambda }.$$

Note that, for the same uncertainty in emissivity, the temperature uncertainty increases with increasing wavelengths. 

**Figure 2:**
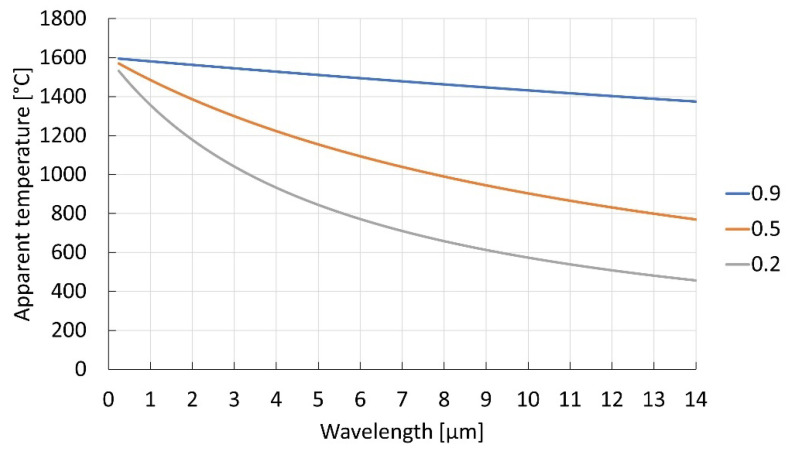
Apparent temperature of an object with a true surface temperature of 2000 K depending on wavelength for three different emissivity values ε.

From Equation (2), we can plot the apparent temperature as a function of wavelength and emissivity (see Figure 2). Note that the temperature difference between the apparent and the true temperature increases with longer wavelength and lower emissivity. Thus, at extremely long wavelengths, a low-emissivity object can appear to be quite cold. The apparent temperature difference for objects of different emissivities will be significant at all wavelengths and highest at the longer wavelengths. 

.

**Figure 3:**
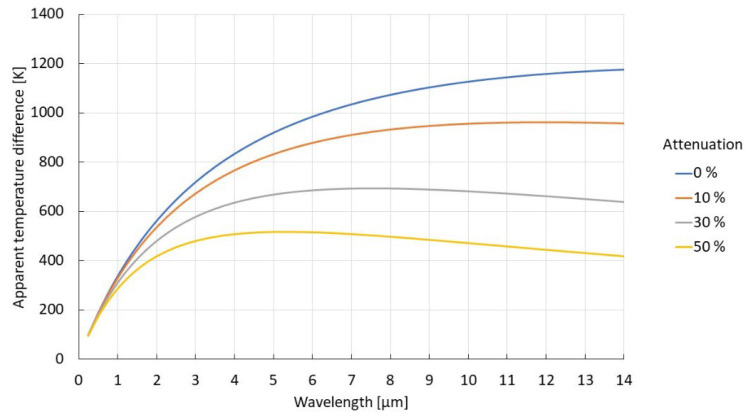
The effect of attenuation along the line of sight on the apparent temperature difference for two objects of different emissivities ($\varepsilon_{1}=0.9$ and $\varepsilon_{2}=0.2$) and the same true surface temperature of 2000 K.

The effect of attenuation along the line of sight on the apparent temperature difference between two objects of different emissivities can readily be understood from the same equation since it affects the received radiance linearly, just as the changing emissivity (see Figure 3). Note that the effect on the difference of the apparent temperature measurements will be significantly smaller at shorter wavelengths than at long ones. This becomes especially important when we are attempting to discern between different materials based on the difference in the apparent temperature, e.g., detection of slag in molten metal streams. While the apparent temperature difference will be largest at longer wavelengths, the influence of any attenuation factors along the line of sight will give the exact opposite effect. In the systems operating at longer wavelengths, variations in the apparent temperature difference will be large, almost in direct proportion to the amount of attenuation: of the order of hundreds of degrees, even for a 10% change in attenuation. In the shorter wavelength region, the difference in the apparent temperatures may be smaller to start with but will be in return relatively unaffected by any changes in the line of sight, exhibiting a very small variation in the temperature difference: of the order of a few degrees, even in case of strong attenuations.

### 2.2. Surroundings Interference and Surface Emissivity Effects

**Figure 4:**
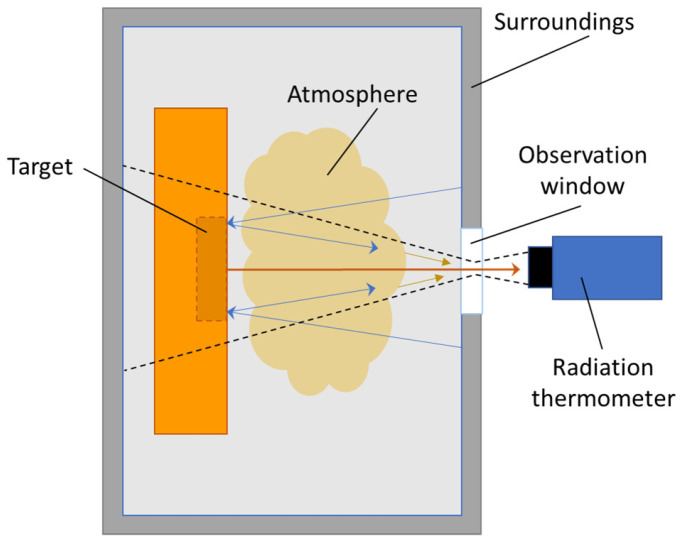
The figure illustrates the different components of the radiation received by the thermometer. Ambient temperature compensation is important when the target does not fill the thermometer’s field of v completely and especially if the target is cooler than the surroundings.

Not all the radiation received by the detector comes from the target. In practice, the total radiation received by the detector comes from the emission of three possible sources: the target, the surroundings, and the atmosphere. The radiation coming from the surroundings can reach the detector through scattering and reflection from the target surface but also directly if the targeted object does not fill the thermometer’s field of view completely (see Figure 4). 

This effect is especially important when the object’s surroundings are at the temperature higher than the target or have an emissivity considerably larger than the target, e.g., in the case of measurements inside furnaces. This is especially pronounced in case of specularly reflecting targets, such as molten metal, but can be compensated for mathematically by a separate temperature measurement of the furnace walls. Finally, the third component of the radiation reaching the detector is the emission from the atmosphere itself. Furthermore, the atmosphere can attenuate the radiation signal along with any other obstacles along the line of sight, for example, clouded observation windows or absorption of the window material itself. For each specific application, this must be evaluated and compensated for. Thus, in order to calculate the temperature of the object correctly, we must know the target object’s surface properties, the temperature of the surroundings, and finally, the atmosphere’s temperature and attenuation properties. 

To reduce effects of surface emissivity and the target environment, we can attempt to control the irradiance of a target surface by using various techniques. Some atmosphere interferences can be filtered optically. In some cases, further intervention may be necessary to further reduce the influence of smoke and dust in the atmosphere. We can approach blackbody conditions also by mechanically fashioning the object surface into a better or ideal emitter, e.g., making a drill- or screw-hole, a long cylinder, a groove etched on the surface, or a wedge [19]. Creating a cavity in the target surface is the most reliable method to reduce effects of surface emissivity where the method is applicable [20]. Such cavities approximate a blackbody by having a surface that folds on itself, having different parts of the surface irradiate each other, and causing the radiation emitted from each surface element to increase. With continued folding, the surface finally takes the form of a cavity with a small opening opposite the observed surface, e.g., the bottom of a drill-hole. The amount of radiation leaving this opening approaches the blackbody value, and the irradiance detected by the instrument increases. The local apparent emissivity of such a blackbody cavity varies at each point along the inside of the cavity walls and depends largely on the intrinsic emissivity of the cavity material and the geometry of the cavity [21]. When the cavity is isothermal, and the aperture radius is small relative to the other cavity dimensions, the effective emissivity is very close to unity, and the cavity approximates a blackbody well even if the surface emissivity of the material it is fashioned from, $\varepsilon_{s}$, is not high. The apparent emissivity of such cavities, $\varepsilon_{a}$, is defined as the ratio of the radiance leaving the surface to blackbody radiance at the same temperature. In case of a reasonably isothermal cylindrical cavity, the apparent emissivity is given approximately by

(4) $$\varepsilon_{a}\approx 1-\left(1-\varepsilon_{s}\right){\left(\frac{R_{aper}}{L}\right)}^{2}$$

where $R_{aper}$ is the radius of the aperture at the front of the cavity, and L is the total length of the cavity [22]. Note that the apparent emissivity increases with the surface emissivity and the length-to-diameter ratio. If $R_{aper}/L\geq 6$, the cavity approximates a blackbody reasonably well independently of the emissivity of the material of which it is made [23]. 

Long, purged sighting tubes can be extended almost to the target. This minimizes the need for cleaning the lenses and windows and also reduces unwanted background radiation [18]. In many cases, closed-end target tubes made of different materials can be used to eliminate the influence of unknown or changing surface emissivity and atmosphere interference [24,25,26]. These are typically used on liquids or in other processes where the inside temperature of the target tube sufficiently represents the process temperature. When a radiation thermometer views the inside of such a cavity, the effective emissivity that it sees is a weighted average of the local effective emissivities of the cavity walls within the thermometer’s field of view. This integrated emissivity will vary from thermometer to thermometer and will vary for a single thermometer if its focal point is changed—for example, by moving the thermometer so that it is focused either on the cavity aperture or on the cavity base. Furthermore, the local effective emissivity of the cavity walls is also affected by any temperature non-uniformities along its length. When a significant temperature gradient exists along the cavity, we must consider the effect of temperature gradients in order to evaluate the target temperature correctly.

When a small temperature gradient exists on the inter-irradiating surfaces comprising a cavity, the apparent emissivity can be approximated to the first order of the local temperature difference [20]. For Wien’s approximation, the apparent emissivity is given by

(5) $$\varepsilon_{a}\left(\lambda \right)\approx \alpha_{a}\left(\lambda \right)+\frac{hc}{\lambda kT}\frac{\Delta T_{e}}{T}.$$

It is approximately equal to the apparent absorptance of the cavity $\alpha_{a}\left(\lambda \right)$, equivalent to the apparent emissivity of the corresponding isothermal cavity, plus a correction factor dependent on the wavelength, temperature, and an effective temperature difference $\Delta T_{e}$. The effective temperature difference is determined only by the geometry, the surface radiative properties, and the temperature distribution of the surfaces. In practice, long target tubes can seldom be assumed to be isothermal. Normally, the top of the target tube is at a considerably lower temperature than the tube’s bottom viewed by the radiation thermometer because of radiative heat losses. When a target is heated on its backside, temperature gradients will be set up along the cavity. The temperature difference between the cavity bottom and the exposed surface will be more pronounced for poorer thermal conductors, higher temperature levels, and longer cavities. Changing temperature gradients will introduce a non-gray effect and thus introduce an uncertainty in the radiometric temperature measurement. 

### 2.3. Radiation Ratio Thermometry

In cases requiring contact-free radiation thermometry where the object surface cannot be mechanically fashioned, the lack of reliable emissivity data is the greatest weakness [27]. It may not be sufficient to use a literature value for the emissivity of the material because the emissivity can vary widely from specimen to specimen with the surface angle, roughness, oxidation level, material grain size, and porosity but also temperature, wavelength, measurement direction, and atmosphere conditions. Measurement in two wavelength regions can be used to eliminate the effect of unknown emissivity of graybodies. Although this implies increased measurement complexity, this type of thermometer can be significantly more accurate than single-band thermometers in many applications. 

The radiation ratio thermometer measures the ratio of the spectral radiances within two narrow spectral bands, centered at λ_1_ and λ_2._ The instrument is calibrated against a blackbody to indicate the same value when the blackbody temperature is $T_{r}$, i.e., the ratio temperature. The true surface temperature can be expressed in terms of the ratio temperature:

(6) $$\frac{1}{T_{s}}=\frac{1}{T_{r}}+\frac{\ln\left(\frac{\varepsilon_{\lambda_{1}}}{\varepsilon_{\lambda_{2}}}\right)}{\frac{hc}{k}\left(\frac{1}{\lambda_{1}}-\frac{1}{\lambda_{2}}\right)}.$$

For the special case when $\varepsilon_{\lambda_{1}}=\varepsilon_{\lambda_{2}}$, that is, for a graybody, it follows that the surface temperature is equal to the ratio temperature. When measuring temperature of a graybody, although it emits less energy at each wavelength than the blackbody, both signals are equally diminished, so the ratio remains unchanged. The radiation ratio thermometer thus measures true temperature for graybodies. Furthermore, the target need not be totally gray; it must just have equal emissivities in the two wavelength regions used by the thermometer. Any non-gray effects can be accounted for by adjusting the relative emissivity, also referred to as the slope of the radiation ratio thermometer, $\varepsilon_{\lambda_{1}}/\varepsilon_{\lambda_{1}}$. Wavelength selection is generally accomplished by optical filtering, commonly termed two-color pyrometry. Additionally, dual sandwich detectors can be employed where the detector pair is monolithically formed on the same substrate, and the top detector is transparent to the radiation to which the lower detector is sensitive. This is commonly termed dual-wavelength pyrometry. 

Any other “gray” effect, that is, an effect equally affecting the two received signals, can be eliminated in the same way. This is, for example, any change in the target surface size as well as changes in the attenuation along the line of sight for targets obscured by smoke or a dusted observation window. The analysis remains the same as long as the obscuring medium is not spectrally selective in its attenuation of radiation, at least in the wavelength regions used by the thermometer. In this case, the temperature inferred by the ratio radiation thermometer remains unaffected by the obstruction. The method can be used even for the targets smaller than the field of view of the thermometer, assuming that the background is cool enough to neglect its thermal radiation. However, significant errors can be made by neglecting thermal radiation from the background as the background temperature approaches that of the target. Similarly, the temperature of the medium occluding a target becomes important as it approaches that of the target. 

Radiation ratio pyrometers measuring both in a monochromatic and two-color mode can be used for direct measurement of unknown emissivity of opaque gray bodies [28,29]. Since the apparent emissivity can be affected by many parameters, for any given material, the emissivity should be measured directly under realistic conditions. In a two-color mode, pyrometers directly measure the relative intensity of the emitted radiation in two narrow bands and relate this ratio to the temperature of the source. Once the temperature has been measured in this way, emissivity can be determined by using the pyrometer in the monochromatic mode and changing the instrument’s emissivity value setting until the measured temperature in the monochromatic mode matches the measured temperature in the two-color mode. If the surface considered is a diffuse gray surface, the emissivity is independent of the viewing angle and wavelength, so the measured value will be equal to the total hemispherical emissivity of the surface. The proposed method was used to investigate the effect of surface finishing on the emissivity values of SiC and graphite at temperatures up to 2000 °C [28]. A decrease of emissivity with surface finishing has been confirmed for polished and unpolished graphite samples as expected, thus pointing to the validity of the proposed method. Moreover, the thermal emissivity of tungsten for high-temperature applications, such as lighting components or heating elements, has been measured by this method [29]. The surface temperature has been accurately determined and unwanted oxidation identified as the main source of measurement error. 

### 2.4. Multiwavelength Radiation Thermometry

As discussed previously, an unknown or changing target emissivity is a critical source of uncertainty. In theory, the limitation of two-wavelength systems to gray surfaces can be overcome by increasing the number of measuring wavelengths. One of the simplest ways of eliminating effects of uncertain emissivities is suggested from the Equation (2):

(7) $$\frac{1}{T_{a}\left(\lambda \right)}=\frac{1}{T_{s}}-\frac{\lambda k}{hc}\ln\left(\varepsilon_{\lambda }\right).$$

The apparent temperature, $T_{a}$, measured by a single-band radiation thermometer at λ approaches the true surface temperature, $T_{s}$, as λ approaches zero [30]. Radiation measurements obviously cannot be made at zero wavelength, but we can measure apparent temperatures at other wavelengths and then extrapolate these results to zero wavelength. Application of multiwavelength thermometry to non-gray targets is limited by this analytical extrapolation process being inherently inaccurate when extrapolating information gathered at relatively long wavelengths to zero wavelength. Even for three wavelengths, the analytical expressions become cumbersome. Nevertheless, a three-band thermometer can measure true temperature where the logarithm of target emissivity depends linearly on wavelength. In general, if this dependence can be expressed as an nth-degree polynomial, a thermometer of n+2 wavelengths can measure true temperature. On the basis of the above discussion, multispectral pyrometry has been developed to accurately determine the temperature of a heated surface in case of unknown emissivity. The application of this method to determining the true temperature for all emissivity behaviors has been limited by the ability to measure at sufficient number of wavelengths to achieve useful accuracies in the extrapolation to zero wavelength. 

In 1979, least-square multi-color pyrometry was first utilized in a three-color pyrometer that determined the temperature of a flame assumed to have constant emissivity [31]. Soon after, the first analysis assuming a non-constant emissivity was published as a computer simulation of a six-color pyrometer. Various multiwavelength systems have since been proposed, all based on the same principle. The emissivity is modelled as a function of wavelength with adjustable parameters to be obtained empirically. The radiation emitted by the target is measured at multiple channels having different spectral characteristics, either monochromatic or wide band. Narrow bands far apart from each other and in the lower wavelength regions seem to provide more accurate solutions [32]. The analysis results in a system of equations whose solution is the target temperature and the parameters of the emissivity function. Saunders presented a method for compensating the effects of background radiation reflections, concluding that the uncertainties in multiwavelength systems may be larger than for single-wavelength pyrometry and suggesting that the use of multiwavelength pyrometry may even become impracticable [33]. Cassady et al. achieved a reduction of the uncertainty in the predicted temperature well below that of single-color pyrometry by adding complementary measurements by using adjacent pixels on a CCD array or including measurements taken at successive times [31]. Many processing techniques have been proposed, strongly relying on the relationship between emissivity and wavelength. Even though several accurate temperature and/or emissivity measurements have been reported, no technique has been universally accepted. The main issue was the large errors arising from an inappropriate selection of the emissivity function but also other sources of uncertainty, such as the effects of the background radiation and fluctuation of the target temperature. This altogether made multi-color pyrometry a rather challenging problem. 

Recently, a new trend of developing intelligent measurement devices has been attracting interest among metrology specialists [34]. Systems can have different design characteristics, but they all employ supplementary functions for maintaining measurement quality. Such systems have the intelligence defined as the ability to keep a good state of metrological operability as long as possible during the impact of changing destabilizing factors. As of today, research into application of artificial intelligence (AI) methods in multiwavelength pyrometry is still scarce. Back-propagation neural networks have been applied to realize automatic recognition of the linear and non-linear emissivity models based on a variety of training sample models [35]. This approach eliminated the need to make assumptions about the emissivity model, thus improving measurement accuracy. Multiwavelength pyrometry combined with an expert system, namely a knowledge representation and reasoning AI approach, has been shown to be able to overcome many of the previously described difficulties of pyrometry [36]. The instrument is comprised of a spectrophotometer and the usual optics. However, it relies on no prior knowledge but examines the data and makes decisions on how to process these data based on the results of the tests that it applies. The approach involves a mathematical representation of the spectral data and uses selected wavelength/intensity pairs to calculate the corresponding temperatures through a radiation ratio thermometry method. If the results are single-valued, as determined by the size of the standard deviation of the ensemble of temperatures, it indicates that the sample is a graybody and that absorptions are not significant. If the standard deviation of the temperatures calculated is large, it indicates one or more of the following conditions: emissivity is a function of wavelength, and atmospheric absorptions are present; the temperature is not single-valued; the optical elements have changed characteristics. Each measurement is analyzed to return both the temperature, the tolerance as a real-time measure of accuracy, and the signal strength value as a quantity directly related to the emissivity at a chosen wavelength. Successful performance has been demonstrated, with accuracy to 0.1% routinely achieved despite emissivity changes, atmospheric interference, and random noise even on materials with changing, spectrally dependent emissivities such as solid and liquid metals.

## 3. Thermal Imaging

Even with the promise of correct temperature and spectral emissivity obtained through application of AI methods, multi-color pyrometers can still be used only for point measurements. When the temperature distribution on the target’s surface is of more interest than absolute temperature point values, e.g., for the detection of local temperature differences such as hot or cold spots, infrared thermography (IRT) or thermal imaging is employed. Such imaging can even reveal material defects by employing active heating by a pulsed heat source, e.g., a halogen lamp or a laser, based on the fact that such defects will readily influence the heat flow following the application of a step heating [37]. In this technique, known as active IRT, the surface temperature increases, and decay is monitored over time to reveal surface cracks and even defects hidden under the object’s surface. The early IRT cameras were optical scanning devices using rotational mirrors and complex electronics to create a two-dimensional (2D) thermal image using a single detector [38]. The complexity of the scanning devices has since been reduced by removing the rotating mirror and applying linear detectors arrays. Nevertheless, these early point sensor or linear array sensors with optomechanical scanners were notorious for their relatively short mean time between failures. The real breakthrough came at the turn of the century when complete two-dimensional detector arrays, comprised of typically many thousands or even several million detectors, called focal plane arrays (FPA) and requiring little to no cooling, became available. FPA cameras, where such detector arrays were placed in the focal plane of an imaging system, offered much longer-lived and more robust equipment. Offering sensitive and fast detection at reduced cost, such cameras continuously gained attention because of many military and civilian applications [39]. 

### 3.1. Thermal Broadband Imaging

FPAs can be based on both photodetectors and thermal microbolometer detectors. Today, bolometer matrixes operating at MWIR and LWIR wavelengths are widely used, providing broadband visible to far infrared imaging and detection capability. The price of FPA based thermal imaging cameras increases dramatically as the operating wavelength increases, especially if cooling is required. Less expensive uncooled thermal detector-based arrays can be used at longer wavelengths at the expense of lower sensitivity and lower speed. The traditional users of thermal imaging are normally not familiar with using shorter, NIR wavelengths. Thermal cameras operating at shorter wavelengths are restricted to use on objects at temperatures above 600 °C [38]. However, as has been explained in Section 2.1, for thermal imaging of high-temperatures targets, multiple reasons deem application of NIR imaging more advantageous than using traditional LWIR thermography, anchored in the fundamental laws of radiation physics. The radiation intensity is maximum at NIR wavelengths, more than an order of magnitude larger than in the LWIR range. The effect of attenuation on temperature measurements will be significantly smaller at shorter than at longer wavelengths. Finally, at shorter wavelengths, low-cost, high-resolution devices such as CCDs can be used [40]. Their spectral response extends into the near infrared region and enables one to easily detect thermal radiations from objects with temperatures higher than 800 °C. When compared to a conventional IR FPA-based imaging, CCD sensors are relatively inexpensive and more affordable to industry. 

**Figure 5:**
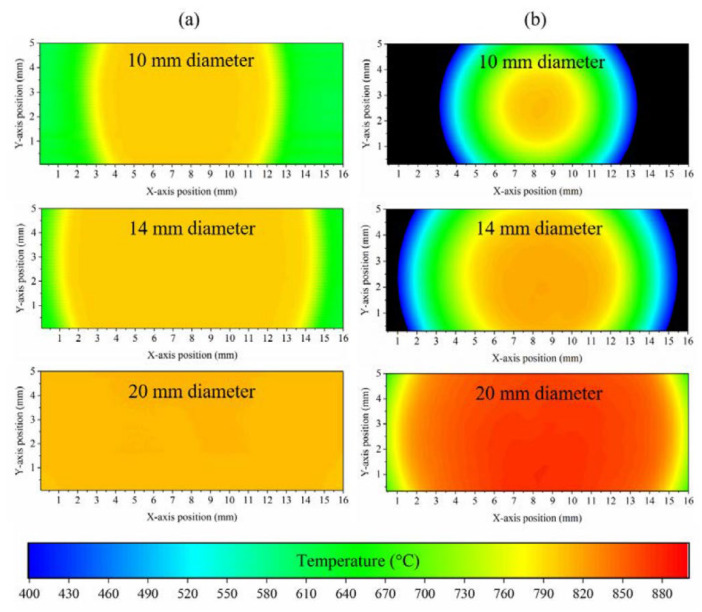
SSE analysis for (**a**) Si APD and (**b**) bolometer camera imagers with increasing target aperture size at a furnace temperature of 800 °C. Reproduced from [42] under license CC BY 4.0.

Designed for visual use rather than precise measurement, FPA-based thermal imaging cameras have disadvantages compared to quantitative radiation thermometry when measurement of target temperature with low uncertainty is required. They can provide qualitative temperature maps to distinguish between different gradients of temperature. However, away from the conditions in which the imager was calibrated, accurate temperature measurements, that is, quantitative thermal imaging across the target surface with defined or quantifiable uncertainty, can be difficult to achieve. The issues with employment of conventional thermal imaging cameras are well-known but unfortunately not well-publicized. They are caused by non-uniformities across the array, both in terms of within the semiconductor material, electronic noise, or the ambient temperature. It is difficult to calibrate a matrix of pixels as identical radiation thermometers. Under the precise methodology in which they were calibrated, they will provide accurate temperature measurement; however, away from those experimental conditions, the conventional thermal imagers provide only indicative temperature measurements. Furthermore, FPAs exhibit unwanted transfer of signals, that is, leakage of photons and electrons between pixels. Such pixel-to-pixel crosstalk can affect the spatial resolution of the detector and thus complicate the reconstruction of the desired image [41]. Another major contributor to this problem is size-of-source effect (SSE), which arises due to imperfections within the optical system, leading to reflections and scattering inside the instrument [42]. Measurements are affected by the power received from outside the field of view, leading to increased measurement uncertainty since each pixel receives power over a wider area of the target than the camera was designed for. The infrared temperature measurement thus becomes dependent upon the size of the target or objects next to the target. How increasing aperture size affects the temperature measurements of a commercial bolometer thermal imagers is shown in Figure 5. To reduce uncertainty in the temperature measurement, instead of FPAs, Hobbs et al. proposed a micromechanical systems (MEMS) mirror-based high-speed 2-dimensional raster scanning in combination with a single-pixel Si avalanche photodiode detector (APD) with an effective wavelength of 1 μm [42]. To demonstrate the benefits of this imaging approach for quantitative thermal imaging, comparison was made with a commercial bolometer-based FPA thermal imager. The cameras were focused onto the same target aperture placed in front of a blackbody calibration furnace set to 800 °C. Although the imaging data for the bolometer camera provided faithful spatial information of the size of the target apertures, there was a large difference in the measured temperature when moving from the center to the periphery. The SSE problem with the FPA was more pronounced with increased target aperture size. For the 20 mm aperture, the temperature measured by the FPA was 876 °C, corresponding to 3.5% of the dynamic range. This is considerably more than that of the Si APD single-pixel change of 10 °C. Furthermore, high internal gain of the Si APD also enabled the measurement of target temperatures below 700 °C with measurement uncertainty of 0.5 °C.

### 3.2. Thermal Spectral Imaging

**Figure 6:**
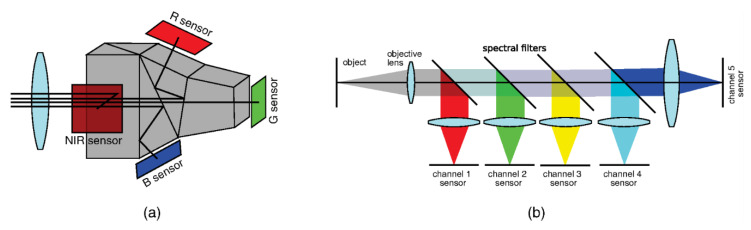
Different multispectral system layouts can be employed based on beam splitting using (**a**) monolithic prism blocks or (**b**) a sequence of spectral filters. Reproduced from [43] under license CC BY 3.0.

Traditional thermographic systems require known or accurately modelled target emissivity conditions. However, application of wavelength sensitive photodetector arrays makes it possible to adopt radiation ratio or multiwavelength radiation thermometry approaches for imaging systems. This enables quantitative thermal imaging also of non-gray targets. Multispectral beam-splitting design can be employed to equally divide the incident energy coming from the target between multiple sensors, as illustrated in Figure 6 [43]. Subsequent multi-sensor fusion enables two-dimensional high-temperature measurements on surfaces with spatial temperature gradients.

Several multispectral imaging systems operating in the visible-infrared spectrum range were proposed for measurement of temperature distributions on high-temperature targets based on CCD [40,44,45], CMOS [46,47], and uncooled MWIR PbSe photodetectors to ensure better coverage of the dynamic range [48]. However, while one can change the beam-splitter designs to adjust the measured spectral bands, it is not easy to divide the incident light into more than four beams without compromising the system performance. Thus, this approach is, in practice, limited to maximally six spectral channels. 

**Figure 7:**
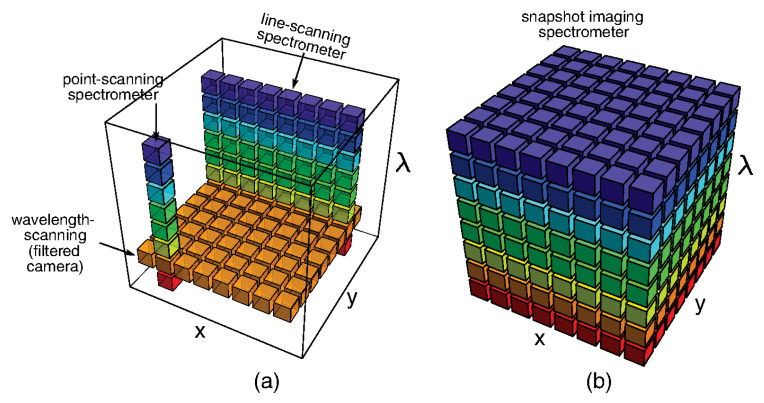
The portions of the datacube collected during a single detector integration period for (**a**) scanning and (**b**) snapshot devices. Reproduced from [43] under license CC BY 3.0.

However, novel spectral imaging sensors have a capability to sample the spectral radiance of a scene $I\left(x,y,\lambda \right)$ and collect a three-dimensional dataset (a datacube). Different systems have been designed to either measure 2D slices of the cube sequentially in time (scanning) or simultaneously measure all elements of the datacube by dividing it into multiple 2D elements that can be recombined into a cube in postprocessing (snapshot) (see Figure 7) [43]. The concept has been in use for many years in several different domains, in the food industry for quality assurance and sorting applications, in medicine for tissue analysis, as well as in remote sensing as a powerful tool for classification of soil and vegetation. Application of such imaging sensors has the potential of extending the measurement capabilities also to uncontrolled scenarios with unknown values of emissivity [49,50,51,52]. This approach offers the advantage of rapid and accurate measurements and capability of scanning large areas in a short time, providing high-quality, real-time data streams [43]. Further developments in the field are expected, driven by the requirements of high-temperature operation, multispectral imaging, lower cost, and higher resolution [53].

The variety of different systems designed to collect spectral imaging datacubes and terminology used interchangeably can be a source of confusion. Spectral imaging is often termed imaging spectrometry (or imaging spectroscopy), hyperspectral imaging, and multispectral imaging. Whiskbroom imaging scans the sample point by point, while pushbroom imaging acquires full spectral information for each pixel in the line. Staring imagers stare down on a sample with a two-dimensional camera to obtain an image. Distinction is often made between systems using few contiguous spectral bands (multispectral imaging) and systems using many and often hundreds of spaced spectral bands (hyperspectral imaging or imaging spectrometry) [54]. Wavelength selection in the scanning systems can be achieved at the source side by illuminating the sample at a specific wavelength or at the detector side by analyzing the reflected light in front of the detector with fixed bandpass filters (e.g., rotating wheel or electronically tunable filters). If the system architecture includes spatial and/or spectral filters, high throughput cannot be assumed [55]. When imaging a dynamic scene, that is, a scene that shows significant spatial and/or spectral change during the measurement period of the instrument, the obtained image can be blurred. 

**Figure 8:**
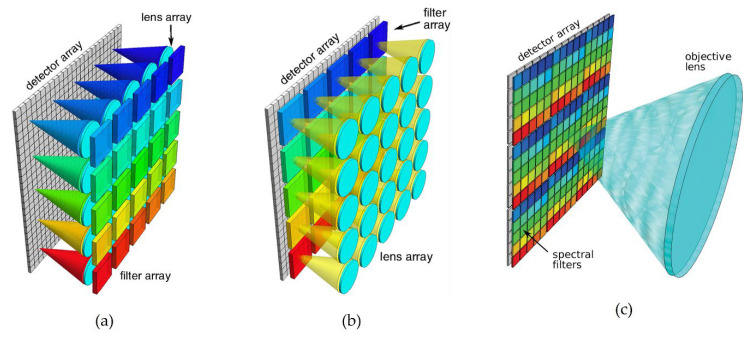
Example of layouts for a multi-aperture-filtered camera system, using (**a**) the Shogenji design and (**b**) IMEC design. (**c**) The system layout for a pixel-level color filter array camera. Reproduced from [43] under license CC BY 3.0.

In contrast, snapshot systems can sense an entire multispectral data cube at one discrete point in time, obtaining the entire dataset during a single detector integration period, thus providing new functionality and extending the domain of spectral imaging towards dynamic, video-rate applications [43]. Modular snapshot imaging systems are based on two or more apertures sensitive to different but close spectral bands, typically 3–15. Multi-aperture-filtered cameras use an array of imaging elements, such as an array of cameras or a monolithic lenslet array, with a different filter placed at each element in order to collect portions of the full spectral band (see Figure 8a,b). The development of Bayer filter-array cameras enabled spectral filtering at the single-pixel level (see Figure 8c). 

## 4. Molten Material Stream Monitoring

As discussed in the introduction, monitoring of the pouring stream during furnace tapping presents a possibility to gain insight into the conditions in the furnace heart. In this section, we will review and discuss the advantages and limitations of the methods proposed for measurement of the different key properties. 

### 4.1. Detection of Slag Carry-Over

In the furnace heart, the molten metal and slag layers segregate into two separate layers due to density difference. During the tapping, molten metal and slag can sometimes be expected to flow out simultaneously from the taphole. In many applications, it is necessary to predict the maximum rate of withdrawal of a fluid with desired properties that can be attained before fluid from a different level also begins to flow. Controlling and limiting the slag carry-over, that is, the mass of steelmaking slag tapped with the steel, is a primary requirement for clean steel production [56]. To maximize the withdrawal rate during tapping the desired fluid, it is necessary to detect when the fluid from a different level begins to flow. The fact that slag and metal exhibit very different emissivity properties can be exploited to separate between the two based on the large apparent temperature difference. For human vision, using a welding glass filter, the visual contrast between slag and metal in the observed scene is small. It requires considerable training for an observer to distinguish between the two, and the method thus involves substantial subjectivity. Alternatively, electromagnetic early-warning systems can be used to detect the slag carry-over, featuring sensors installed in the tapping zone between the steel casing and the refractory lining [57]. However, this entails considerable shortcomings related to installation on the vessel itself, often requiring modifications to the taphole area to ensure reliable operation. The major disadvantage is the durability problem, as the wiring of the equipment runs through the rotary transmission of the converter vessel or electric arc furnace. 

As an alternative to conventionally used human visual detection or sensors installed on the vessel itself, slag detection systems have been developed based on LWIR microbolometer FPAs [57,58,59]. Such a slag detection system can offer continuous operation with low maintenance, automatic triggering of alarm at the end of tapping, and minimally interfering contact-free operation. LWIR exploits a wavelength range where the difference in emissivity between slag and molten metal is highest (8–14 µm). This results in slag apparently having significantly higher temperature than metal when seen by the LWIR thermal imager. The general layout of such slag detection systems is rather simple, involving a camera positioned to capture the full view of the pouring stream. The harsh environment can seriously affect the expensive LWIR infrared camera and its sensitive lens, requiring a protective environmental enclosure with an air-cooling system. LWIR transmissivity must be considered since quartz windows are opaque to LWIR wavelengths. Images can be acquired continuously and differentiation between metal and slag made by online digital image processing. 

**Figure 9:**
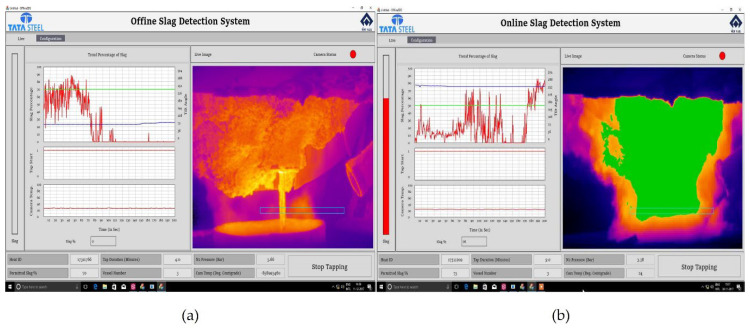
(**a**) LWIR image of the tapping stream while steel is pouring out of the furnace. The temperature of the stream was about 1650 °C. (**b**) Under flames and fumes condition, the images captured by the LWIR camera can be quite noisy, and any measurement made on these data will be useless [57].

The position of the pouring stream will change during the different phases of tapping, and any movement must be accurately tracked. An automatic tracking method that can quickly find the edge was proposed by Zhang et al., together with a calibration method for the temperature threshold to distinguish the molten steel, calculating the amount of slag in real time [58]. Limiting the measurement to the area identified as the stream reduces errors that may be caused by background heat sources in the field of view. Patra et al. used the information about the furnace tilt angle available from the system and defined a corresponding rectangle in the image through which the stream has to pass [57]. In this case, all the pixel values within the boundary of rectangle were considered and marked slag or steel based on pre-set temperatures, and percentage slag within stream was calculated. Avoiding unnecessary calculations allowed manifold increase in the detection process speed, thus improving detection time and time constant of the overall system, analyzing the molten stream to detect the slag percentage automatically in real time. However, a considerable challenge has been identified related to false alarms arising due to fumes and flames (see Figure 9). 

This observation can be readily explained by the influence of varying and unpredictable amount of attenuation along the line of sight having a much more pronounced influence in the LWIR region than at shorter wavelengths, as discussed in Section 2.1. During furnace tapping, a great deal of visual attenuation can be expected to occur because of clouds of smoke rising from the ladle but also dirt accumulation on the optics. It is difficult to predict accurately the amount of attenuation likely to be encountered, making this the key factor of influence on the measured apparent temperature difference. Under such conditions, the images captured by the LWIR camera can be quite noisy. At shorter NIR wavelengths, the attenuation along the line of sight will have a significantly smaller effect on apparent temperature measurements. A successful development of cost-effective NIR thermal imaging systems for generating thermographs of hot objects at temperatures above 350 °C using Silicon CCD detector arrays was already reported in 1995 [60]. Several interesting examples of their use were reported, including the detection of iron in a slag stream runner based on the ability to detect the brightness differences, a use very similar to the already described LWIR slag detection systems. 

### 4.2. Measurement of Surface Temperature

Temperature of molten metal streams is commonly measured using pyrometers. However, unknown or changing surface emissivities of molten metals present a major challenge. To calculate true temperature, some knowledge about the normal spectral emissivity as a function of temperature is required. Typical metallic behavior corresponds to increase in the normal spectral emissivity with temperature [61]. A modified Hagen–Rubens relationship can be used to obtain good estimates for the normal spectral emissivity of liquid metals and alloys at temperatures up to 1000 K above their melting points if their electrical resistivity is known as a function of (radiance) temperature in the liquid phase [62]. Note that this does not apply to measurements in the solid state since the emissivity of a material does not only depend on its intrinsic optical properties but also on their surface texture. However, in case of molten metals, any surface oxides that might be present on the surface of a solid sample can be assumed to evaporate by the time the sample reaches temperatures that exceed the melting point. Above melting, surface tension is believed to rapidly smooth the surface of the liquid sample Thus, the molten metal surface can be expected to be almost perfectly specular, and any radiation coming from the surroundings will be able to reach the pyrometer by means of reflection. This effect is especially important when the surroundings are at the temperature higher than the target or have an emissivity considerably larger than the target, and this must be taken into consideration in thermal imaging of the tapping stream. On top of this, a varying position of the pouring stream can make a pyrometer measurement virtually impossible since the pyrometer spot target must accurately match the stream of molten material. 

**Figure 10:**
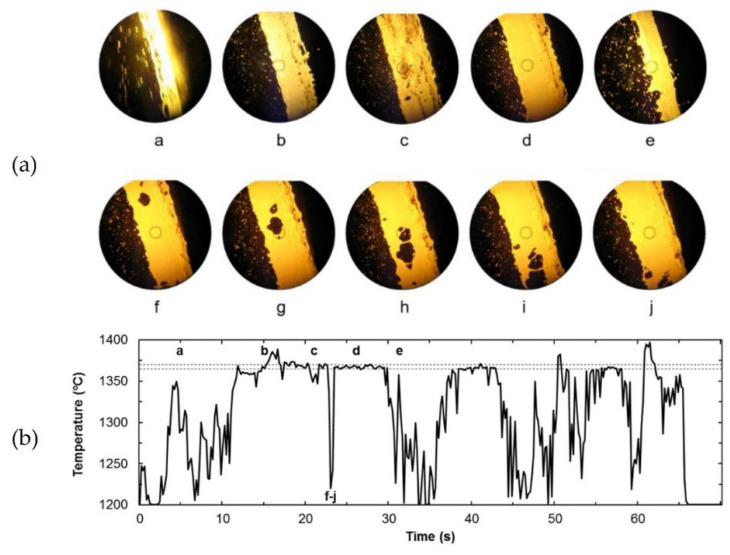
(**a**) The image presents selected video frames showing relevant features of the hot metal stream affecting the radiation thermometer measurement. The first row: narrow shape with high slag content; liquid slag with bright streaks; solid slag spots; almost clean stream with hot metal exposed; narrow shape with liquid slag streaks. The second row: shallow stream with a hole produced inside. The circle in the center indicates the location of the radiation thermometer measurement spot, less than 100 mm in diameter. (**b**) Radiation thermometer output obtained during the pouring of 150 t of hot metal. Letters a–j correspond to the typical stream features illustrated in the panel (**a**). Reproduced from [63] under license CC BY 4.0.

In order to understand the behavior of the hot metal stream and identify the causes of signal fluctuation and design the processing of the pyrometer signal, Diaz et al. proposed a multi-sensor approach combining IR thermometry with a simultaneous digital video camera recording [63]. A simple procedure for hot metal temperature forecasting from available existing plant data was presented to assess the feasibility of infrared thermometry as an early estimator of actual temperature of tapped hot metal from a blast furnace in the oxygen steel-making process. The different measuring and modeling approaches were found to be complementary, and combined methodology of indirect measurement and statistical forecasting was proposed for providing the best available hot metal temperature prediction to be used by the charge model. Combining measuring and modeling approaches reduced the error to 13 °C with 100% reliability, thereby providing a hybrid procedure that has long-term stability and is self-adaptive to varying production scenarios. A radiation thermometer operating at 550 nm wavelength and 1200 to 1700 °C temperature range was used. Video recording allowed identification of the most common causes of pyrometer signal fluctuation and perturbation, thus elucidating under what circumstances the IR signal provided better information (see Figure 10). 

Recognizing the challenges related to using pyrometers on molten material streams, Usamentiaga et al. investigated the use of infrared thermography for temperature measurement of molten pig iron [64]. The proposed system confronted the challenges of the position and size of the stream in the images changing with the angle of the pivoted torpedo car and the slag partially covering the molten pig iron stream. A LWIR camera was used to obtain infrared images of the stream. In order to measure the molten metal temperature in a stream partially covered by slag, the pixels imaging slag were removed by thresholding based on the difference in the apparent temperature measured, followed by region growing. Special attention was given to the calibration procedure since the accuracy of an infrared temperature measurement system highly depends on correctly adjusted emissivity. To measure the temperature accurately, emissivity had to be estimated under the same conditions as the temperature measurement. The authors proposed to use a lance with a thermocouple immersed in the molten pig iron inside the torpedo to measure the reference temperature needed for the emissivity calibration. Immediately after the temperature measurement procedure with the lance, the molten pig iron inside the torpedo car was poured into the ladle, the stream was imaged, and the emissivity in the infrared camera changed until the median of the temperature distribution matched the temperature measured previously with the lance. However, this temperature measurement procedure is potentially dangerous, as it requires direct human intervention in the vicinity of the hot material. Furthermore, the temperature measured by the infrared camera had a delay with respect to the temperature measured with the lance. The temperature of the molten pig iron could change during this period of time. Nevertheless, the proposed method produced more consistent measurements when compared to the conventional pyrometer. This was understood to be mainly due to the difficulty of always matching the pyrometer spot target to the molten metal stream. 

**Figure 11:**
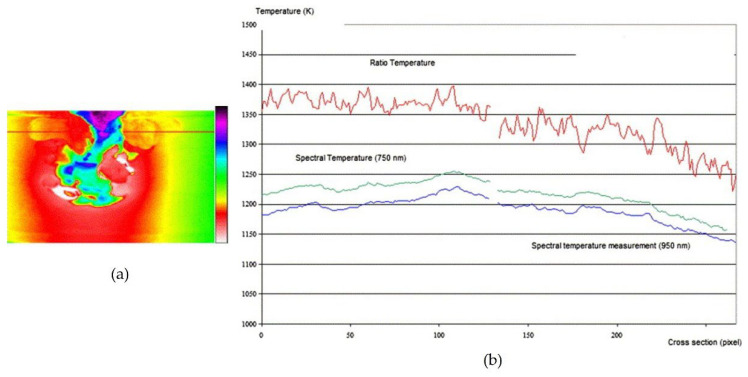
(**a**) Thermal image of a copper sample heated up to its fusion point. (**b**) Temperature profiles along the red horizontal line. The red curve was obtained through radiation ratio method, while the green and blue curves were obtained with spectral temperature method at 750 nm and 950 nm, respectively [40].

Meriaudeau proposed a real-time dual-wavelength imaging system for control applications in industry related to surface treatment processes such as quenching, laser heating, laser cladding, laser welding, etc. The system was comprised of two CCD cameras [40]. The incident energy coming from the target was equally divided between the two cameras via a beam splitter. Each of the two cameras imaged the target through an interference filter centered at 750 and 950 nm. Simultaneous temperature measurements at different wavelengths at a rate of 25 frames per second could be performed. Figure 11 presents the obtained temperature profiles for a sample of copper that has been heated by electromagnetic induction. The two-wavelength method, which assumes the graybody behavior, gave an accurate result, whereas the spectral temperatures were wrong due to the lack of information about the emissivity. 

The two-color method was also applied for the detection of combustion in the blast furnace tuyere raceway to obtain the two-dimensional projection temperature distribution [65]. The radiation images of the flame in the raceway were obtained using a color CCD camera. The two wavelengths could be arbitrarily selected from the camera’s red, green, and blue channels. Two-dimensional temperature distributions of the raceway were reconstructed at average temperatures about 1956 °C. The developed temperature measurement system could accurately evaluate the combustion situation in the raceway, thus contributing to the stable operation of the blast furnace. 

Moreover, it is possible to exploit the same setup and use a method involving imaging at three wavelengths to overcome the effect of the unknown emissivity when the emissivity can be described as a linear function of wavelength. A multispectral synchronous imaging pyrometer, operating in the visible–infrared spectrum range, was developed to measure the temperature distribution on a wing leading edge in an extreme plasma aerodynamic heating environment [45]. The system was based on the beam-splitting design and multi-sensor fusion of three CCD sensors with different monochromatic filters inserted in front to give to give three spectral measurements. The filters were centered at 700 nm, 810 nm, and 920 nm. The temperature measurement uncertainty was within 0.21 °C–0.99 °C in the temperatures of 600 °C–1800 °C for the blackbody measurements, implying the high measurement accuracy of this pyrometer at the standard experiment conditions. The experiments verified the advantage of the three-color pyrometry for 2D measurements of surfaces with large spatial temperature gradients. To simultaneously measure two-dimensional temperature and emissivity distributions on high-temperature diffuse surfaces, a method to simultaneously measure two-dimensional temperature and emissivity distributions on high-temperature diffuse surfaces using an auxiliary light source was proposed [66]. The optical pyrometer system was based on a color CCD image sensor and a hemispherical-distributed quartz lamp array. The high-temperature diffuse surface was irradiated from with the auxiliary light source, and two images of the effective radiation intensity were obtained in quick succession as the lamp was switched “on” or “off” to determine the temperature and emissivity distributions. The method was found very useful for accurately measuring the temperature distribution, accurately compensating for the effect of unknown or changing emissivities at high temperatures of 600–1000 °C. The reported temperature measurement uncertainties were less than 4 °C. 

Jirasuwankul proposed that approximate thermal imagery of glowing hot objects could be reconstructed from images in RGB color space by a well-trained artificial neural network model [67]. Decoupled images of the glowing hot object to individual monochromatic red, green, and blue channels were fed as multiple inputs to the proposed model. Experimental results showed that averaging error of the estimated temperature could be achieved with 10% for the reddish-yellowish hot objects and less than 10% for the bright-yellow ones. 

To broaden the temperature range that could be measured, a four-color imaging pyrometer was developed to investigate the thermal behavior of laser-based metal processes, specifically laser welding and laser additive manufacturing of stainless steel [46]. The pyrometer was targeted to span 600–3500 °C. The proposed instrument consisted of four, high-sensitivity silicon CMOS cameras configured as two independent two-color pyrometers combined in a common hardware assembly. This coupling of pyrometers permitted low- and high-temperature regions to be targeted within the silicon response curve, thereby broadening the useable temperature range of the instrument. Bandpass filters at 450 nm and 500 nm were selected for measurements at high temperatures, while 850 nm and 950 nm filters were chosen for low-temperature measurements. Wójcik et al. demonstrated the use of an external bandpass filter and a combination of signals generated by an RGB CMOS matrix to detect thermal radiation simultaneously in three overlapping spectral regions [47]. It was shown that it is necessary to ensure the stability of the solution of the system of three nonlinear equations with respect to the influence of noise. For this purpose, the use of a priori information about the slope factor of the emissivity in the selected spectral range was proposed. The proposed solution allows creation of inexpensive thermographic equipment for monitoring and control of complex thermal processes.

To ensure better coverage of the dynamic range, PbSe sensors can be employed. An uncooled snapshot five-color multispectral imaging system was developed to enable quantitative thermal imaging in the MWIR range independent of emissivity. Medium-resolution images (128×128 px) were acquired at high-speed rates (2000 fps) [48]. Compared to cooled alternatives that work in this spectral range (e.g., InSb or HgCdTe), this approach offers a low-cost solution and a small format that enables the design of affordable and compact multi-aperture solutions. Different apertures and different spectral bands were built by using five filters centered at 2.25, 2.75, 3.5, 4.25, and 4.75 μm wavelengths, all with 0.5 μm spectral width. Unlike previous works on two-color and multicolor thermography, based on the use of the ratio of responses between pairs of close spectral bands or approximations of the Planck’s law, the authors adopted a flexible and transparent approach to modeling multiple regression based on machine learning and using synthetic datasets. High accuracy and robustness against variations in the value of emissivity were demonstrated. To estimate the capability of the proposed system to deal with non-gray bodies, the authors simulated this behavior as a local variation of emissivity in the spectrum. The system could handle local variations of emissivity of up to a 20% in the spectrum of one of the bands, with an acceptable impact on the measurement accuracy. This work demonstrated a simple and compelling approach to robust and reliable thermographic imaging regardless of emissivity. The system was tested in different application scenarios (e.g., firefighting and industrial monitoring) with promising results. The proposed approach offers significant benefits through its modularity and reusability. Apertures can be added or redesigned with different off-the-shelf optical components to provide accurate tailored systems.

**Figure 12:**
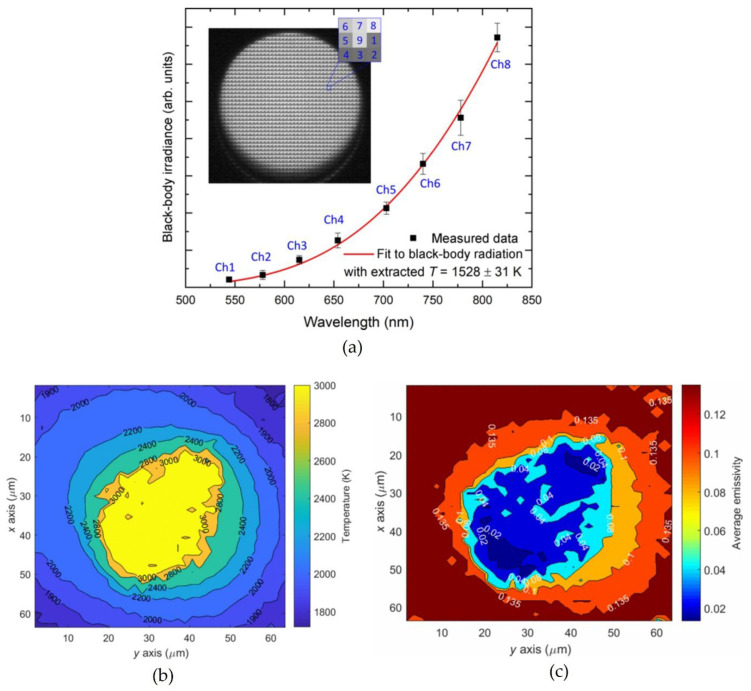
(**a**) Spectral radiance measured by the hyperspectral camera when imaging a known blackbody source cavity at a temperature of 1473 K. The experimental data fit well with the prediction based on Planck’s law for blackbody radiation. Inset: raw image of the blackbody source cavity consisting of a matrix made up of groups of 3 × 3 spectral-band-filtered pixels called macropixels. An enlarged view of the nine interleaved pixels in a macropixel is shown in the top right corner. (**b**) Temperature-profile measurements during laser heating. (**c**) Emissivity profile measurements during laser heating [51].

Spectral imaging sensors have only recently been employed for high-temperature measurements. In 2017, Devesse et al. presented a system based on a hyperspectral line camera to capture the spectra in the visible and near-infrared (VIS/NIR) region applied to temperature measurement of liquid stainless steel [49]. This system resulted in a temperature profile of the melt pool surface with a very high spatial resolution, suitable for applications where high-temperature measurements with high spatial detail are desired, such as in the laser material processing and additive manufacturing. The main advantage compared to classical camera-based systems was that a complete spectrum was available at each location so that the emissivity could be estimated at each point. A total of 512 spectral lines from 400 nm to 950 nm were acquired at a frame rate of 1000 frames per second. Measuring the spectral radiance at a number of uniformly spaced wavelengths $\lambda_{i}\;\left(i=1,\;\ldots ,\;N\right)$ resulted in a set of measurements that include some additional measurement noise. A more general description of the emissivity as a linearly decreasing function of wavelength was adopted, as found appropriate for some liquid metals in previously published literature [68,69]. Assuming that the noise is zero-mean and Gaussian distributed with known variance, the maximum likelihood estimates of the temperature T and the parameters of the emissivity function could be obtained by solving the nonlinear least-squares problem. In general, there is no guarantee that this solution corresponds to the true temperature. Without a perfect knowledge of the spectral emissivity variation, the true temperature value could only be determined within a range bounded by a lower and an upper value. Measurements of a liquid melt pool of stainless steel showed that the system was able to determine the absolute temperatures with an accuracy of 10%. Hyperspectral imaging has also been employed to measure the distribution of temperature and emissivity of a candle flame [50]. Radiation images were taken with a hyperspectral imaging system with an internal scanning mechanism. Spectral range was from 372.5 nm to 1038.6 nm with 4.68 nm resolution (128 spectral bands) with the speed of 30 spatial lines per second. The authors proposed a Newton-type iterative method to solve the polynomial coefficients of radiative parameters and wavelength. The radiation characteristics of the candle flame were found to differ from a graybody, and the emissivity of the flame was found to decrease as the wavelength increases. Qu et al. proposed a multispectral imaging method to capture the spectral radiance in eight spectral bands and obtain surface temperature measurements during laser-based materials processing [51]. The application of traditional thermography is challenging in this case since the emissivity may be changing constantly in the laser-material interaction region, where the temperature gradients are extreme, and surface displacement can complicate the measurement. Time-resolved temperature measurements during microsecond pulsed-laser irradiation of a metal plate made of titanium alloy provided temperature information with an estimated accuracy of ±10%. The spectral images were captured using an off-the-shelf snapshot multispectral camera operating in the VIS/NIR range made of a commercial 1.3 Mpx CMOS image sensor. A multispectral matrix filter employs macropixels that integrate eight wavelength band-pass filters ranging from 544 to 815 nm and one panchromatic (white light) filter, arranged in a 3 × 3 matrix (see inset in Figure 12a). The spectral radiance emitted by the surface was captured with a spatial resolution of 15.9 µm/pixel and a maximum temporal resolution of 60 frames per second. The temperature and emissivity values were estimated simultaneously by fitting the measured spectral radiance to Planck’s law, assuming that the spectral emissivity decreases linearly with wavelength (see Figure 12). 

A high-speed hyperspectral imaging system has been developed for laser welding application, comprised of a custom designed lens system and a high-speed CMOS camera [70]. A graybody approximation was adopted as valid for liquid steel surfaces and temperature and emissivity measurements obtained in a least-squares regression approach to the spectral data. The use of this setup was investigated also for fiber laser flame cutting application to characterize the temperature and melt flow behavior of the cutting front of mild steel plates [52]. The observed cut-front temperatures were significantly higher than the melting point of mild steel and could reach up to 3200 K. The approach was proven to be a powerful technique for investigating a number of highly dynamic cutting process phenomena. 

### 4.3. Surface Conditions and Material Composition

Emissivity is strongly dependent on the surface conditions, with both surface structure and material composition as important contributing factors. Metals and especially liquid metals are an extreme example, exhibiting substantial changes depending on composition, surface morphology, phase, and temperature. Furthermore, metals usually exhibit a spectral dependence of emissivity that also changes with these characteristics. A typical feature of unoxidized metals is the normal spectral emissivity decreasing with wavelength and increasing with temperature [38,61]. The large volume of data collected through multiwavelength pyrometry indicates that emissivity is dependent on the condition of any given sample [29,68]. Thus, any conventional temperature measurement that depends on an assumed behavior of emissivity is unlikely to be accurate. For radiation thermometry to measure temperature accurately, emissivity must be addressed during each measurement. The monotonic decrease of emissivity with wavelength in metals was found to be common but not universal. While the emissivity of liquid silicon [71] and liquid steel [70] in VIS/NIR range have been reported to be fairly constant, that of titanium has been found to be linearly decreasing with wavelength [69]. Surprisingly small changes in composition have been reported to affect the emissivity greatly. Multiwavelength radiation thermometry measurements in VIS/NIR range were made on rods of pressed tantalum powder sintered in vacuum [36]. A difference in compositions of only a few atomic percent of alloying materials lead to a change in emissivity from graybody to colorful behavior and function of wavelength. Similar studies of new and old tungsten filament lamps revealed that the spectral dependence of emissivity changed with age, starting as spectrally dependent and finally becoming practically gray [68]. This was found to be due to evaporated tungsten deposits on the bulb being re-deposited on the filament, while the bulk property resistivity governing temperature vs. current remained unchanged. Multiwavelength pyrometry performed on nickel superalloys melted in an induction-heated vacuum investment casting furnace revealed that emissivity changed significantly with both phase, turbulence, and composition [68]. The emissivity decreased more than 60% with the phase change from solid to liquid state. The power settings were changed manually several times and an abrupt, transient increase in emissivity has been observed with each power increase. The surface of the melt was disturbed by the violent stirring of the melt resulting from the inductive electromagnetic pulse, and the resulting rough surface exhibited a higher emissivity. As the melt returned to the previous behavior, the emissivity value settled back as well. 

**Figure 13:**
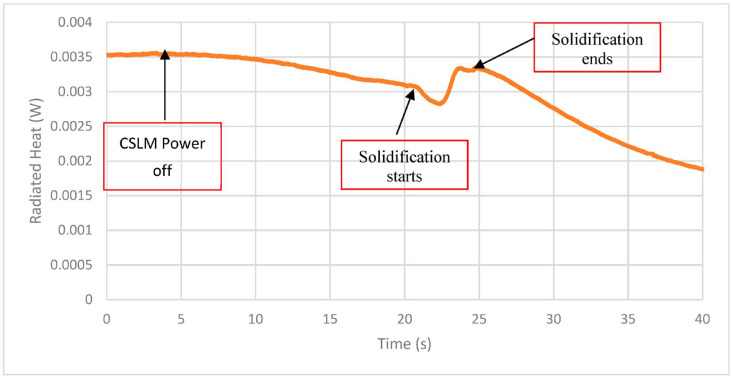
Radiated heat curve for the surface of the steel sample that was solidified in argon. Reproduced from [72] under license CC BY 4.0.

Slater et al. explored the potential of using LWIR thermography for surface characterization during solidification of twin-induced plasticity steel under different gas atmospheres to detect and differentiate changes resulting from reactions (formation of surface films) and/or phase changes (solidification) due to the differences in emissivity and latent heat [72]. In order to induce physical changes resulting from phase transitions and chemical reactions on the liquid steel surface and during solidification, controlled changes in temperatures and/or gas atmosphere were used. In this work, LWIR thermography was used during solidification in argon, carbon dioxide, and nitrogen atmospheres using a confocal scanning laser microscope (CSLM). It was found that surface reactions resulted in a solid oxide film (in carbon dioxide) and decarburization, along with surface graphite formation (in nitrogen). In both cases, the emissivity and hence the cooling rate of the steel were affected in distinct ways. Differences in nucleation conditions (free surface in argon compared to surface oxide/graphite in carbon dioxide/nitrogen) as well as chemical composition changes (decarburization) affected the liquidus and solidus temperatures, which were detected by thermal imaging. It was noted that the temperature readings taken from the surface acted to provide information on when events occurred rather than providing a direct temperature reading itself (see Figure 13). Once the power of the heating was turned off, a gradual decrease in the radiated heat could be seen due to the cooling of the sample. After around 20 s, the sample emissivity dropped quickly due to convection ripples on the liquid surface due to the formation of solid subsurface. The following increase could be seen, which was attributed to both the latent heat of fusion as well as the higher emissivity that the solid surface exhibited in comparison to the melt. After solidification, the slope of the radiated heat was steeper than prior to solidification, suggesting the higher emissivity was resulting in a greater cooling rate of the sample.

### 4.4. Surface Flow Rate

Analysis and control of fluid flows, often subsidiary to industrial design issues, require measurements of the flow field [73]. To study the flow field and flow pattern in casting molds, water model experiments can be carried out. However, the multi-phase flow behavior and related phenomena in air–water systems are quite different from that in molten metal systems. Therefore, it is highly desirable to measure the velocity of molten metal directly [74]. Few solutions for opaque liquids are available commercially. Metallic and semiconductor melts pose additional problems involving high temperature and chemical aggressiveness. It has been deemed practically impossible to measure the turbulence characteristics in molten metal flows even at a temperature of around 200 °C [75]. Although some kinds of velocimeters have been developed for molten metal flows, their accuracy was not high, and accordingly, previous numerical results of molten metal flow velocities in actual processes such as ladles did not receive any experimental confirmation. Due to the harsh process conditions, contactless methods are preferred. Ratajczak et al. presented a short review about the recent developments of measurement techniques focusing on the development of contactless inductive measurement techniques exploiting the high electrical conductivity of those melts [76]. These measurement techniques include the contactless inductive flow tomography, which is able to reconstruct the mean three-dimensional velocity structure in liquid melts; local Lorentz force velocimetry, which enables the local assessment of flows close to the wall; and inductive methods for bubble detection. 

The velocity of flow on the surface of a molten metal bath can be measured through imaging of tracer particles on the liquid surface and subsequent application of particle image velocimetry methods such as multiple exposure technique, consecutive time step images, or binary image cross-correlation method, assuming that flow velocities in the flow field do not change suddenly in time and space [75]. Recognizing two-color imaging in the visible spectrum as a good alternative to more classical imaging based on high-cost IR thermal cameras, Andreu proposed a dual-wavelength system for quality assessment of steel processing based on CCD cameras [44]. During the steel production, the flow pattern of the slag is a visible indicator of the process quality. Traditionally, slag motion is observed visually on an irregular basis by an operator who interprets its motion. Motion can be specified as “good” if the direction of the homogeneous slag is flowing towards the electrode from all directions or “bad” if the slag flows from the center of the electrode towards the border of the mold. The proposed system allowed a real-time, contactless monitoring of the slag temperature (with an accuracy of ±5 °C) and a continuous monitoring of the flow patterns based on motion tracking. The estimated graybody temperatures were reported with a maximum error of 0.5% of the experimental temperature range. To satisfy the graybody assumption, the two wavelengths were chosen sufficiently close to each other while at the same time being distant enough to allow sufficient sensitivity of the instrument. In an image sequence, the moving patterns of the slag caused temporal variations of the image brightness, and continuous monitoring of the flow patterns of the ingot slag topping could be realized based on motion tracking. Assuming that all temporal intensity changes were due to motion only, analyzing the slag motion was performed based on calculating how much each image pixel moved between adjacent images. 

**Figure 14:**
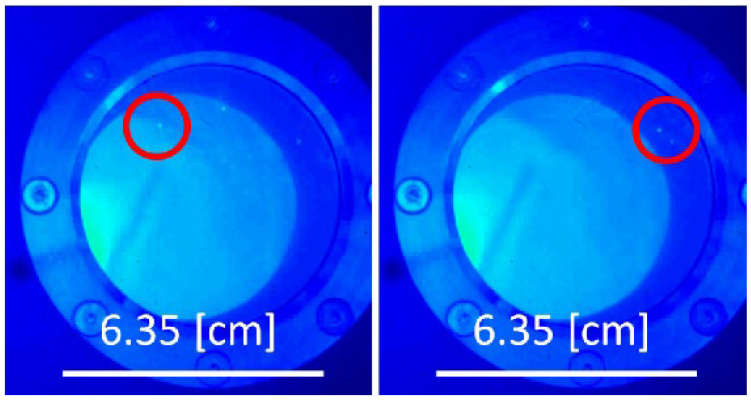
A sample of data collected at consecutive timestamps using the MWIR camera and the particle tracking method. MWIR compatible windows were installed above the free-surface flow. The impurity particle used as a tracer is marked by a red circle [77].

Hvasta et al. presented a MWIR camera-based method of measuring the surface velocities of open-channel liquid-metal flows for liquid-lithium plasma-facing components in fusion reactors [77]. A particle tracking technique was employed to monitor the flow of liquid metal alloy, which was liquid at room temperature. Small amount of impurities, developing and floating on the metal surface, were used as tracers during this experiment (see Figure 14). Tracer imaging was based on the thermal and optical differences between the matte oxides and the mirror-like liquid metal. The data were used to better understand open-channel flows and determine surface boundary conditions. To validate the data collected using the particle tracking technique, the surface velocity measurements were compared to the bulk velocity measurements, yielding similar results. The authors noted that surface velocity measurements could not be assumed to correspond to bulk fluid velocities without further research. This method could be implemented and automated for a wide range of liquid–metal experiments even if they operate at high-temperatures or within strong magnetic fields. 

## 5. Conclusions 

A comprehensive review of possibilities and limitations of different thermal imaging methods for monitoring high-temperature molten material has been presented in this paper, together with the information on the radiation thermometry working principles. Applicability for monitoring of the pouring stream for understanding, optimalization, and control of furnace tapping processes has been discussed. Commercially available thermal cameras operating in the MWIR and LWIR regions are found to be more suitable for applications requiring better dynamic range coverage and qualitative assessment of thermal gradients rather than for accurate quantitative measurements. For quantitative thermal imaging of metals at temperatures higher than 600 °C, operation in VIS/NIR domain offers advantages such as radiation intensities more than an order of magnitude larger than in the LWIR range. Furthermore, the effective emissivities of molten metal surfaces can be expected to be highest at shorter wavelengths. Glass is transparent at these wavelengths, so quartz windows and inexpensive glass optics with good imaging capabilities can be used. For slag carry-over detection systems based on the measurement of the apparent temperature difference, operation at VIS/NIR wavelengths entails significantly improved robustness to any variations in the amount of attenuation along the line of sight caused by changes in the atmosphere or accumulation of dirt on optics and access windows. At shorter wavelengths, the apparent temperature difference may be smaller to start with but is in return virtually unaffected by any changes even in case of strong attenuations. In the systems operating at longer wavelengths, while the apparent temperature difference will be largest, any variations in the line of sight will cause changes in the apparent temperature difference: of the order of hundreds of degrees, even for a 10% change in attenuation. Finally, at shorter wavelengths, low-cost, high-resolution CCD or CMOS devices can be used. When compared to conventional thermal imaging systems, such sensors are more affordable to industry. Furthermore, the detection is based on photonic effects and is therefore wavelength-sensitive, with response times in the order of microseconds, enabling adoption of multiwavelength radiation thermometry approaches and realization of quantitative thermal imaging on non-gray, varying emissivity targets. Spectrally resolving detector arrays enable multi- and hyperspectral imaging with compact instruments robust to perturbations such as temperature changes and vibration. High-speed hyperspectral imaging has been demonstrated for investigation of highly dynamic process phenomena at temperatures as high as 3200 K. Further developments in the field are expected, driven by the requirements of high-temperature operation, lower cost, and higher resolution. Based on the fundamental properties of light, spectral imaging methods have the potential to generate vast amounts of high-quality data in real time. Combined with artificial intelligence methods for real-time analysis and integration of expert knowledge, spectral imaging can indeed offer more robust and accurate measurement of high-temperature molten material stream properties such as surface temperature, composition, turbulence, and flow rate.
